# Abundance of DNA adducts of 4-oxo-2-alkenals, lipid peroxidation-derived highly reactive genotoxins

**DOI:** 10.3164/jcbn.17-90

**Published:** 2017-12-12

**Authors:** Yoshichika Kawai, Erika Nuka

**Affiliations:** 1 Department of Food Science, Graduate School of Biomedical Science, Tokushima University, Kuramoto-cho 3-18-15, Tokushima 770-8503, Japan

**Keywords:** lipid peroxidation, aldehyde, DNA adduct, 4-oxo-2-alkenal

## Abstract

Reactive oxygen species and their reaction products can damage DNA to form mutagenic lesions. Among the reactive species, lipid peroxidation-derived aldehydes react with nucleobases and form bulky exocyclic adducts. Many types of aldehyde-derived DNA adducts have been characterized, identified and detected *in vitro* and *in vivo*, whereas relative quantitative and pathophysiological contributions of each adduct still remain unclear. In recent years, an abundant class of DNA adducts derived from 4-oxo-2-alkenals have been identified, in addition to classic aldehyde-derived adducts. The presence of 4-oxo-2-alkenal-derived DNA adducts associated with age-related diseases has been revealed in rodents and humans. *In vitro* studies have demonstrated that 4-oxo-2-alkenals, as compared with other classes of lipid peroxidation-derived aldehydes, are highly reactive with nucleobases. It has been generally recognized that 4-oxo-2-alkenals are generated through oxidative degradation of the corresponding 4-hydroperoxy-2-alkenals, homolytic degradation products of polyunsaturated fatty acid hydroperoxides. Our recent results have also shown an alternative pathway for the formation of 4-oxo-2-alkenals, in which 2-alkenals could undergo the metal-catalyzed autoxidation resulting in the formation of the corresponding 4-oxo-2-alkenals. This review summarizes the basis of the formation of lipid peroxidation-derived genotoxic aldehydes and their covalent adduction to nucleobases, especially focusing on the abundance of 4-oxo-2-alkenal-derived DNA adducts.

## Introduction

**Fig. 1:**
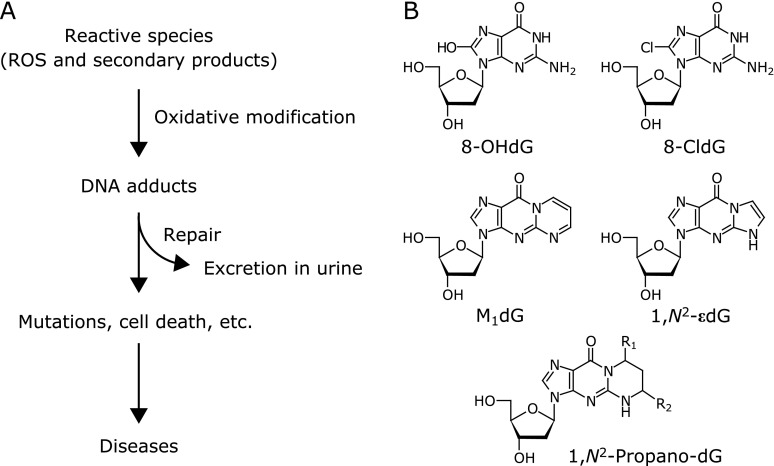
Oxidative DNA modification by ROS and their secondary products. (A) Scheme for oxidative DNA modification and its consequences, if not repaired, associated with various diseases. (B) Representative well-known 2'-deoxyguanosine (dG) adducts formed by ROS, reactive halogen species, and lipid peroxidation products. 8-OHdG is a well-studied, major oxidative DNA product formed mainly by the reaction with hydroxyl radical. 8-chloro-2'-deoxyguanosine (8-CldG) is a chlorinated DNA product formed by the reaction with hypochlorous acid. Three types of lipid peroxidation-derived dG adducts are also illustrated. Malondialdehyde, 2-alkenals and epoxyaldehydes (formed by peroxide-mediated oxidation of 2-alkenals) react with dG to form M_1_dG, 1,*N*^2^-propano-dG (R_1_ = OH, R_2_ = alkyl or R_1_ = alkyl, R_2_ = OH), and 1,*N*^2^-etheno (ε)-dG, respectively.

Covalent modifications of DNA bases by various exogenous and endogenous compounds have been implicated in the process of carcinogenesis and various diseases.^(1–3)^ Although DNA adducts are normally repaired and excreted in urine in order to maintain the fidelity of the DNA, if the lesions were not repaired, subsequent DNA replication can lead to mutations or apoptosis (Fig. 1A).^(4,5)^ Mutations in protooncogenes and tumor suppressor genes have been implicated in cancer,^(6)^ cardiovascular diseases and neurodegenerative diseases.^(7–9)^ Endogenously generated reactive oxygen species (ROS) can attack nucleobases in free nucleotides/nucleosides and DNA/RNA to form oxidatively modified nucleobases, such as 8-hydroxy-2'-deoxyguanosine (8-OHdG, Fig. 1B).^(10,11)^ Reactive nitrogen and halogen species, such as peroxynitrite and hypohalous acids (hypochlorous acid and hypobromous acid), can also modify nucleobases to form nitrated and halogenated products, such as 8-nitroguanine and 8-halogenated-2'-deoxyguanosine (Fig. 1B), respectively.^(12–16)^ Alternatively, ROS can initiate lipid peroxidation reactions, generating a variety of reactive products that react with nucleobases to form adducts.^(4,10,11,17)^ Thus, much attention has long been focused on the relationships between lipid peroxidation and carcinogenesis. Because the processes of lipid peroxidation reactions are quite complex and not fully understood, the elucidation of overall structures and reaction mechanisms of lipid peroxidation-derived DNA adducts has not yet been completed. Recent advances in mass spectrometric analysis, especially liquid chromatography-tandem mass spectrometry (LC-MS/MS), enable us to detect multiple compounds simultaneously. Recently, some researchers have reported the detection of various lipid peroxidation-derived DNA adducts comprehensively using LC-MS/MS, called “DNA adductomics” approach,^(18,19)^ and exhibited the relationship between the amount/pattern of DNA adducts and the risk of several diseases. Through such DNA adductomics, we and other several research groups have focused on the abundant formation of 4-oxo-2-alkenal-derived DNA adducts *in vitro* and *in vivo*.^(19–22)^ This review summarizes the formation of lipid peroxidation-derived DNA adducts, especially focusing on the formation of 4-oxo-2-alkenal-derived DNA adducts.

## Lipid Peroxidation-Derived DNA Damage

**Fig. 2:**
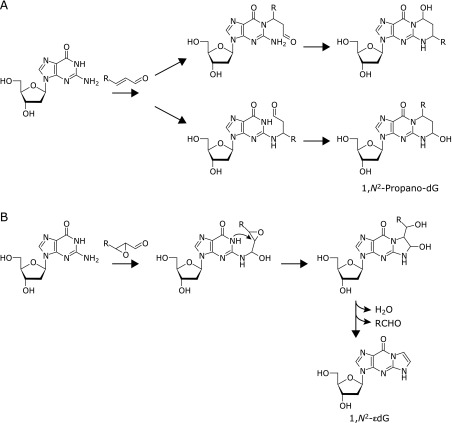
Proposed reaction schemes for the formation of 1,*N*^2^-propano-2'-deoxyguanosine (dG) (A) and 1,*N*^2^-εdG (B).

Lipid peroxidation, one of the results from the accumulation of ROS, termed “oxidative stress”, is considered to be involved in various diseases such as cancer, cardiovascular diseases and neurodegenerative diseases.^(23–25)^ Polyunsaturated fatty acids, such as arachidonic acid and linoleic acid, essential components of cellular membranes and lipoproteins, are the major targets for lipid peroxidation. During lipid peroxidation reactions, lipid hydroperoxides are generated as primary products and then oxidatively degraded to a variety of aldehydes as secondary products.^(26)^ Among numerous lipid peroxidation-derived degradation products, malondialdehyde (MDA), acrolein, crotonaldehyde, 4-hydroxy-2-nonenal (HNE) and 4-hydroxy-2-hexenal have been well-studied in the reactivity to biomolecules such as protein and DNA.^(27,28)^ Especially, HNE and 4-hydroxy-2-hexenal are considered as specific markers for peroxidation of ω-6 and ω-3 polyunsaturated fatty acids, respectively.^(29,30)^ Among the lipid peroxidation-derived degradation products, several classes of aldehydes possess high reactivity against nucleobases, in particular guanine, which is prone to oxidative modifications under physiological conditions. Formation of 1,*N*^2^-substituted cyclic 2'-deoxyguanosine (dG) adducts with various aldehydes have been characterized and identified.^(31–33)^ The most widely studied exocyclic adducts are the propano-, etheno (ε)- and MDA-derived dG adducts (Fig. 1B). The propano-adducts are formed from α,β-unsaturated aldehydes or enals, such as acrolein, crotonaldehyde, and HNE.^(32,33)^ The propano-dG adducts are formed by Michael addition of *N*-1 of dG to C-3 of the α,β-unsaturated aldehydes, followed by ring closure between *N*^2^ of dG and the C-1 aldehyde group. The propano adducts are also formed through the ring closure in the opposite direction (Fig. 2A). The ε-adducts are products of reactions with epoxides of enals.^(34,35)^ The ε-adducts are formed as follows. Attack by the exocyclic amino group of the nucleoside on the carbonyl carbon of the epoxyaldehyde, followed by cyclization via nucleophilic attack on the internal carbon of the C2 epoxy group by *N*-1 and H_2_O elimination, yields alkyl-substituted ε-adducts. Further elimination of the carbon side chain leads to the formation of non-substituted ε-adduct (Fig. 2B). Formation of DNA lesions caused by these aldehydes *in vivo* have been revealed in rodents and human tissues by various analytical methods such as mass spectrometry, ^32^P-postlabeling, and immunostaining.^(36–40)^

## Discovery of 4-Oxo-2-Alkenals as a New Class of Lipid Peroxidation Products

**Fig. 3:**
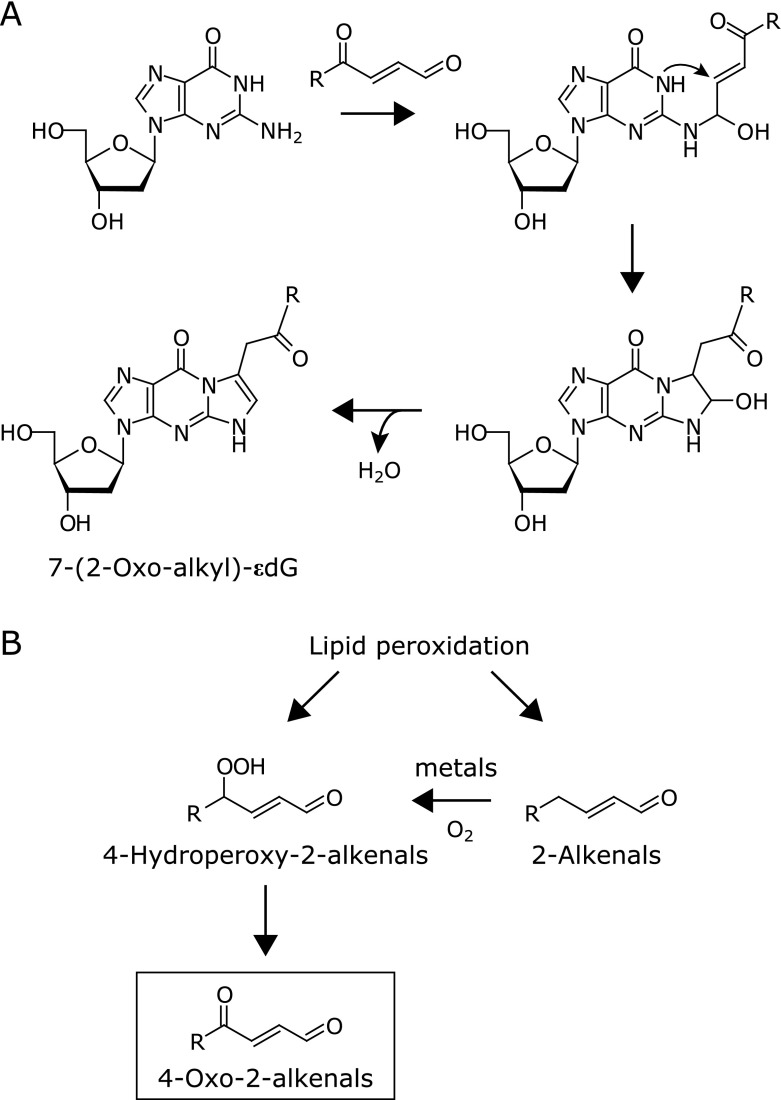
Formation of 4-oxo-2-alkenals and their dG adducts. (A) Scheme for the reaction of 4-oxo-2-alkenals with 2'-deoxyguanosine (dG) to form 7-(2-oxo-alkyl)-εdG. (B) Proposed two pathways of the formation of 4-oxo-2-alkenals during lipid peroxidation. 4-Oxo-2-alkenals are presumed to be formed from the corresponding 4-hydroperoxy-2-alkenals, which are thought to be formed through homolytic degradation of polyunsaturated fatty acid hydroperoxides. It was recently found that metal-catalyzed autoxidation of 2-alkenals also generates 4-oxo-2-alkenals,^(57)^ probably through the formation of 4-hydroperoxy-2-alkenals.

These studies, described above, strongly support that lipid peroxidation-derived reactive aldehydes could be important endogenous genotoxins. However, the relative contribution of different types of aldehydes to the formation of exocyclic DNA adducts has not yet been established. After 1999, in addition to already well-investigated genotoxic aldehydes, Blair’s group has discovered the formation of 4-oxo-2-nonenal (ONE) as a novel lipid peroxidation-derived aldehyde.^(41)^ They have shown that ONE is formed through the decomposition of linoleic acid hydroperoxides and reacts with dG, 2'-deoxyadenosine (dA), and 2'-deoxycytidine (dC) yielding 2-oxo-heptyl-substituted ε-adducts *in vitro* and *in vivo*.^(41–44)^ The reaction mechanism for the formation of ONE-dG adduct is initiated by nucleophilic addition of *N*^2^ of dG to the aldehydic carbon of ONE followed by Michael-type addition reaction between C-2 of ONE and *N*-1 of dG, resulting in the generation of ethano ring. This intermediate readily underwent dehydration to stable 2-oxo-heptyl-substituted ε-adducts (Fig. 3A). Through similar reaction mechanisms to the formation of dG adducts, 4-oxo-2-alkenals could form 2-oxo-alkyl-substituted 3,*N*^4^-etheno-dC and 1,*N*^6^-etheno-dA adducts upon reaction with dC and dA, respectively.^(42,43)^ Our group has immunohistochemically demonstrated, for the first time, the presence of the ONE-dG adduct *in vivo* in a rat carcinogenesis model.^(20)^ These findings suggested that ONE and perhaps other 4-oxo-2-alkenals could be a new class of endogenous genotoxins.

Our group has also developed a monoclonal antibody (mAb6A3) specific to ONE-dG adduct and revealed for the first time the presence of this adduct *in vivo*.^(20)^ Significant immunostaining with mAb6A3 was observed in the liver of rats fed the choline-deficient l-amino acid defined (CDAA) diet. The CDAA diet is known as an experimental model for endogenous rat liver carcinogenesis associated with oxidative stress.^(45)^ It has been shown that 8-OHdG, an established promutagenic oxidative DNA lesion, was significantly increased in livers by the CDAA diet and was involved in the development of putative preneoplastic lesions.^(45)^ In agreement with the increasing lipid peroxidation (thiobarbituric acid-reactive substances) levels, positive staining with mAb6A3 was observed in nuclei of the liver of CDAA-fed rats, but not stained in control groups, showing the formation of ONE-dG adduct in nuclear DNA associated with lipid peroxidation levels. Significant immunopositive staining of ONE-dG was also observed in the spinal cord motor neurons of patients with sporadic amyotrophic lateral sclerosis.^(46)^

ONE is one of the major breakdown products of linoleic acid hydroperoxides.^(41)^ ONE is an analogue of HNE, a representative end-product commonly derived from oxidized ω-6 polyunsaturated fatty acids. It was also found that ONE-dG formation was commonly observed in the DNA incubated with oxidized ω-6 polyunsaturated fatty acids (linoleic acid, arachidonic acid and γ-linolenic acid).^(20,47)^ These results clearly shown that the hydroperoxides of ω-6 polyunsaturated fatty acids are potential sources of ONE and its DNA adducts *in vivo*. In contrast, although ONE-derived adducts were not formed upon reaction with oxidized ω-3 polyunsaturated fatty acids (α-linolenic acid, eicosapentaenoic acid and docosahexaenoic acid),^(20)^ the formation of DNA adducts with 4-oxo-2-hexenal were also observed instead.^(22)^

It has recently been reported that ONE reacts not only with nucleobases but also with nucleophilic amino acids, such as arginine, cysteine, histidine, methionine and lysine residues.^(48–51)^ It is notable that ONE is far more reactive with cysteine and reduced glutathione (GSH), than HNE.^(48)^ GSH, ubiquitously distributed in biological systems, is known to be important in defense systems against oxidative stress.^(52–54)^ The α,β-unsaturated aldehydes react with the sulfhydryl group of GSH via a Michael-type addition reaction, resulting in the formation of covalently adducts and the loss of sulfhydryl groups.^(55)^ Relatively weak mutagenic activity of HNE may be due to its higher reactivity with sulfhydryl groups,^(56)^ rather than nucleobases. GSH also can bind to ONE, as well as HNE, to a greater extent than other α,β-unsaturated aldehydes. These observations suggest that intracellular sulfhydryl groups, especially GSH, could largely contribute to protect DNA bases. Therefore, if the intracellular GSH were depleted under oxidative stress, 4-oxo-2-alkenals and other reactive aldehydes may significantly react with intracellular nucleobases and then play an important role in the endogenous process of carcinogenesis.

## Abundance of ONE-Derived DNA Adducts

**Fig. 4:**
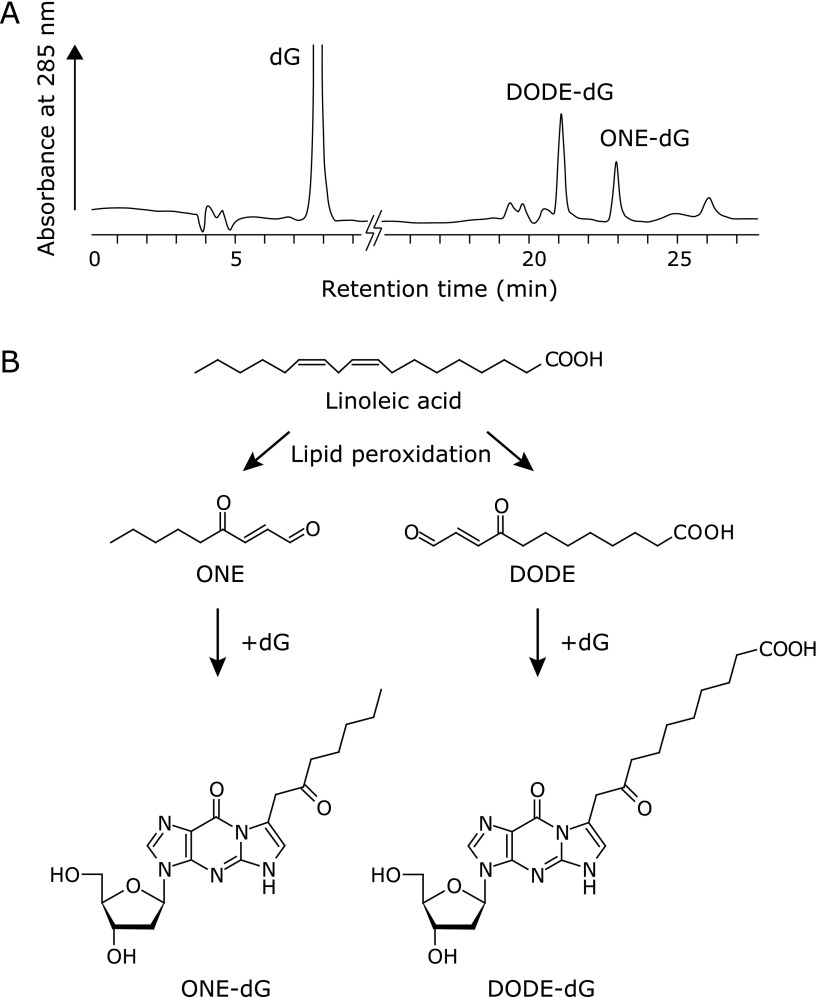
4-Oxo-2-alkenals are highly reactive aldehydes toward nucleobases. (A) Representative high-performance liquid chromatography (HPLC) profile for the reaction mixture of 2'-deoxyguanosine (dG) and linoleic acid in the presence of Fe^2+^/ascorbate, a free radical generation system. This HPLC chromatogram was modified from our previous paper.^(21)^ Two dG adducts with ONE (4-oxo-2-nonenal) and DODE (9,12-dioxo-10-dodecenoic acid) were detected as major products. (B) Proposed scheme for the formation of ONE, DODE and their dG adducts.

To identify the major DNA adducts with lipid peroxidation products, we have previously examined the reactions of each 2'-deoxynucleoside (dG, dC, dA, or thymidine) with oxidized linoleic acid, and then analyzed the products by high-performance liquid chromatography, mass spectrometry, and nuclear magnetic resonance.^(21)^ During incubation with oxidized linoleic acid, dG and dC were significantly decreased and several new products were detected instead. The modification of dA was not significant and no modification of thymidine, which has no exocyclic NH_2_ groups, was observed. It is of interest that the major dG and dC adducts were all derived from ONE or the carboxylic analog 9,12-dioxo-10-dodecenoic acid. As an example, a chromatogram and a proposed scheme for the formation of the major dG adducts are shown in Fig. 4A and B. In addition, higher reactivity of ONE with dG and dC, rather than dA and thymidine, was reproduced *in vitro*.

Abundant formation of ONE-derived adducts could also be explained by the observation that the reactivity of ONE with nucleobases *in vitro *was much higher than other lipid peroxidation-derived aldehydes (acrolein, MDA and HNE).^(21)^ One exception was in the case of glyoxal, which significantly reacted with dG. However, glyoxal-dG adduct was scarcely detected in the reaction of oxidized linoleic acid with dG, suggesting the relatively low amount of glyoxal formed during linoleic acid peroxidation. These results strongly suggested that dG and dC adducts with 4-oxo-2-alkenals could represent the major DNA adducts derived from lipid peroxidation products. To further study the formation of ONE-derived adducts in double-stranded DNA, ONE-derived dG, dC and dA adducts in enzymatic hydrolysates of DNA samples were analyzed using LC-MS/MS. It is of interest that, although all of the ONE adducts were detected in double-stranded DNA treated with ONE, the DNA hydrolysates contained a large amount of ONE-dC adduct compared with the dG and dA adducts. The preferential formation of ONE-dC adduct was also reproduced upon reaction of ONE with single-stranded DNA or 12-mer homo-oligonucleotides. These results suggest that, in contrast to the comparable levels of dG and dC adducts in the free nucleosides, dC residues may represent the major target of ONE and perhaps other 4-oxo-2-alkenals in higher molecular DNA and oligonucleotides. Several research groups have demonstrated, using DNA adductomic analyses, the abundant formation of 4-oxo-2-alkenal-derived DNA adducts *in vitro* and *in vivo*.^(19–22)^ The observation that ONE-dC adduct was indeed detected as one of the major adducts in one human pulmonary DNA could support the *in vitro* studies for higher reactivity of ONE with dC residues in DNA.^(19)^

## New Pathway for the Formation of 4-Oxo-2-Alkenals

The example of discovering ONE raises the possibility that there could still be unidentified lipid peroxidation-derived aldehydes and the DNA adducts. Thus, we investigated the DNA adductomics derived from the Fe^2+^-oxidized arachidonic acid, and then found several unidentified lipid peroxidation-derived dG adducts *in vitro*. We detected a major unidentified adduct by LC-MS/MS at *m/z* 390 → 274.^(57)^ Based on the molecular ion, we speculated that the aldehyde(s) with 8-carbon chain as the reactants. We then analyzed the reaction mixture of dG with several commercially available aldehydes with 8-carbon chain and found that this unidentified adduct was successfully detected upon reaction with 2-octenal. Surprisingly, ^1^H-NMR spectrum of this adduct was similar to those of previously reported ONE- and 4-oxo-2-pentenal-derived dG adducts.^(21,41)^ We then identified this adduct to be 7-(2-oxo-hexyl)-εdG, which is presumed to be formed upon reaction with 4-oxo-2-octenal (OOE). Indeed, this adduct was predominantly formed in the reaction of dG with authentic OOE. This unexpected finding suggested that OOE could be formed during incubation of 2-octenal and dG.

It has been understood that 2-alkenals mainly generate propano-adducts through Michael-type addition. Otherwise, peroxide-mediated epoxidation of 2-alkenals leads to the formation of different types of DNA adducts, etheno-adducts, through epoxide-opening and/or retro-aldol reactions.^(58–60)^ Indeed, Michael-type 2-octenal-dG adducts and 2,3-epoxyoctanal-derived dG adducts were also detected upon reaction with 2-octenal. However, OOE-dG was unexpectedly formed as one of the major products in the reaction of dG with 2-octenal. We also confirmed that other 2-alkenals (with at least 5 carbon atoms) also generated corresponding 7-(2-oxo-alkyl)-εdG adducts, suggesting that 4-oxo-2-alkenals could be formed from the autoxidation of 2-alkenals. Furthermore, we confirmed by LC-MS/MS that OOE itself was indeed produced during incubation of 2-octenal in the presence of transition metals. Thus, we proposed a new pathway for the formation of 2-alkenal-derived DNA adducts, in which 2-alkenals (with five or more carbons) could be oxidized at C4-position into the corresponding 4-oxo-2-alkenals and then react with DNA bases (Fig. 3B). It has been reported 4-hydroperoxy-2-nonenal, a major homolytic degradation product of hydroperoxy ω-6 polyunsaturated fatty acids, undergoes metal-catalyzed degradation into ONE and HNE.^(61)^ Similarly, the formation of OOE could be mediated through 4-hydroperoxy-2-octenal, although the formation of 4-hydroperoxy- and 4-hydroxy-2-octenal has not yet been revealed during autoxidation of 2-octenal.

## Biological Consequences of Lipid Peroxidation-Derived DNA Adducts

DNA damage is thought to contribute to carcinogenesis, aging, and neurodegenerative diseases through mutations, genome instability, and perturbed signaling. Several papers have reported that lipid peroxidation-derived aldehydes and their exocyclic DNA adducts could be implicated in mutations. For example, five-membered exocyclic dG adducts could induce nucleotide misincorporation *in vitro* and *in vivo*.^(62)^ It has also been reported that 2,3-epoxy-4-hydroxynonanal, which reacts with dG generating alkyl-substituted εdG adducts, analogous to ONE-dG, is highly mutagenic in *Salmonella typhimurium*.^(56)^ In addition, the formation of 2-oxo-propyl-εdG, structurally analogous to ONE-dG, was reported upon incubation of dG with 4-oxo-2-pentenal, a hydrolyzed metabolite of *N*-nitrosopiperidine, a carcinogenic cyclic nitrosamine.^(63,64)^ Furthermore, the mutagenic potential of the substituted εdC adduct has also been suggested.^(65)^ The genotoxicity of 4-oxo-2-alkenals has also been directly examined.^(66,67)^ These results strongly suggest that, in addition to other well-investigated aldehydes, 4-oxo-2-alkenals could also be mutagenic aldehydes associated with carcinogenesis.

Researchers have already known that epigenetic changes could be associated with cancer. Importantly, epigenetic changes affecting genetic regulation and cellular differentiation may lead to alterations in embryology, aging, cancer and other diseases.^(68)^ The best known epigenetic modification is cytosine methylation, the role of which is not fully understood. However, it is well accepted that cytosine methylation functions to control gene expression and protects the host organism from expression of undesired sequences.^(69)^ Aberrant DNA methylation patterns, hypermethylation and hypomethylation, have been discovered in many kinds of human cancers.^(70,71)^ At present, we didn’t yet know whether oxidative DNA damage could be associated with epigenetic alteration and cause or consequence of cancer. 8-OHdG is considered the most frequently detected and studied oxidative DNA lesion.^(72)^ The presence of 8-OHdG in oligonucleotides profoundly alters the enzymatic methylation of adjacent cytosines. Therefore, it is possible that increased levels of 8-OHdG reduce cytosine methylation, and influence the carcinogenic process. Moreover, global DNA methylation levels, and levels of oxidative stress markers 8-OHdG and 8-isoprostane were assessed in metal oxide nanomaterial handling workers.^(73)^

In contrast to the only major oxidative DNA lesion 8-OHdG, relatively few information is available on the biological consequences of lipid peroxidation-derived DNA lesions. To clarify the importance of lipid peroxidation-derived DNA modifications in the next future, we have to investigate further comprehensive analysis of major aldehyde-DNA adducts *in vivo*, identify the specific modification sites of lipid-derived aldehydes in DNA, as previously reported,^(74)^ and also analyze their relationships with mutations, cell death, and also DNA methylation.

## Abbreviations

CDAA: choline-deficient l-amino acid defined
dA: 2'-deoxyadenosine
dC: 2'-deoxycytidine
dG: 2'-deoxyguanosine
ε-dG: etheno-dG
GSH: glutathione
HNE: 4-hydroxy-2-nonenal
LC-MS/MS: liquid chromatography-tandem mass spectrometry
MDA: malondialdehyde
8-OHdG: 8-hydroxy-2'-deoxyguanosine
ONE: 4-oxo-2-nonenal
OOE: 4-oxo-2-octenal
ROS: reactive oxygen species
